# Supplementary material for: Antimicrobial Peptide Octoprohibitin-Encapsulated Chitosan Nanoparticles Enhanced Antibacterial Activity against Acinetobacter baumannii
Source: Pharmaceutics. 2024 Sep 25;16(10):1245. doi: 10.3390/pharmaceutics16101245 (PMC11510178; doi:10.3390/pharmaceutics16101245)
Supplement: Supplementary file 1 [file pharmaceutics-16-01245-s001.zip › pharmaceutics-3127798-supplementary.pdf]

Supplementary Table S1. Optimization of encapsulation process, encapsulation efficiency (EE%) and loading capacity (LC%).

	Reaction 1	Reaction 2	Reaction 3	Reaction 4	Reaction 5
CS: CMC: AMP	0.4: 2: 0	0.4: 2: 0.25	0.4: 2: 0.5	0.4: 2: 1	0.4: 2: 1.5
EE%	NA	83.8	83.1	85.7	75.5
LC%	NA	8.7	17.3	34.2	47.2
